# Expanding the Mutational Spectrum of *ACADVL*: Integrative Characterization of the p.Ser72Phe Variant in Very Long-Chain Acyl-CoA Dehydrogenase Deficiency

**DOI:** 10.3390/genes17060649

**Published:** 2026-05-31

**Authors:** Francesca Dinatolo, Lucia D’Antona, Radha Procopio, Valentina Rocca, Elisa Lo Feudo, Samuele Martino, Adele Dattola, Fernanda Fabiani, Emma Colao, Rosario Amato, Francesco Trapasso, Margherita Ruoppolo, Giulia Frisso, Daniela Concolino, Nicola Perrotti, Giuseppe Viglietto, Rodolfo Iuliano

**Affiliations:** 1 Medical Genetics Unit, Renato Dulbecco University Hospital, 88100 Catanzaro, Italy; francesca.dinatolo@studenti.unicz.it (F.D.); dantona@unicz.it (L.D.); valentina.rocca@unicz.it (V.R.); elisa.lofeudo@studenti.unicz.it (E.L.F.); adele.dattola@studenti.unicz.it (A.D.); f.fabiani@materdominiaou.it (F.F.); colaoemma@unicz.it (E.C.); rosario.amato@unicz.it (R.A.); trapasso@unicz.it (F.T.); perrotti@unicz.it (N.P.); viglietto@unicz.it (G.V.); 2 Department of Health Sciences, Campus S. Venuta, University Magna Graecia of Catanzaro, 88100 Catanzaro, Italy; 3 Department of Medical and Surgical Sciences, Neuroscience Research Center, Magna Graecia University, 88100 Catanzaro, Italy; 4 Department of Experimental and Clinical Medicine, Campus S. Venuta, University Magna Graecia of Catanzaro, 88100 Catanzaro, Italy; 5 CEINGE-Biotecnologie Avanzate Franco Salvatore S.C.A R.L., 80145 Naples, Italy; mruoppol@unina.it (M.R.); gfrisso@unina.it (G.F.); 6 Department of Molecular Medicine and Medical Biotechnology, University of Naples Federico II, 80131 Naples, Italy; 7 Pediatric Unit, Department of Medical and Surgical Sciences, Magna Graecia University of Catanzaro, 88100 Catanzaro, Italy; dconcolino@unicz.it

**Keywords:** very long-chain acyl-CoA dehydrogenase deficiency, fatty acid oxidation disorders, *ACADVL*, p.Ser72Phe variant, newborn screening

## Abstract

Background/Objectives: Very long-chain acyl-CoA dehydrogenase deficiency (VLCADD) is an autosomal recessive disorder of mitochondrial fatty acid β-oxidation caused by pathogenic variants in *ACADVL*. The clinical spectrum is highly heterogeneous, ranging from lethal neonatal cardiomyopathy to late-onset myopathy. This study aims to characterize the rare c.215C>T (p.Ser72Phe) variant, identified in compound heterozygosity with the common pathogenic allele c.848T>C (p.Val283Ala) in a male neonate detected by newborn screening (NBS). Methods: Genetic analysis was performed using Sanger sequencing on the proband and his family members. The pathogenicity of the p.Ser72Phe variant was evaluated through multiple bioinformatic predictors and interpreted according to ACMG/AMP guidelines. To understand the functional impact on the protein, structural modeling was conducted using FoldX 4.0 for energy calculations and UCSF ChimeraX for the visualization of conformational changes and cofactor-binding site perturbations in the VLCAD homodimer. Results: At the end of the first postnatal week, liquid chromatography–tandem mass spectrometry (LC-MS/MS) analysis of dried blood spots of the proband revealed a markedly abnormal acylcarnitine profile, with C14:1 levels (1.837 μmol/L) approximately five times above the reference range. Clinical reports documented hypoketotic hypoglycemia, consistent with VLCADD. Segregation analysis demonstrated transmission of both variants within the family, with additional heterozygous and homozygous carriers identified. Bioinformatic predictions uniformly classified p.Ser72Phe as deleterious. This variant has an extremely low allele frequency and affects a highly conserved residue in the FAD-binding domain. Structural modeling with FoldX yielded a mean ΔΔG of +22.63 ± 5.48 kcal/mol, indicating a significant localized thermodynamic burden. Inspection of the mutant model in ChimeraX showed perturbation of the side-chain orientation and attenuation of the local hydrogen-bonding network at the FAD-binding site, together with increased steric packing around residue 72. Taken together, the clinical, genetic, and structural evidence support reclassification of p. Ser72Phe as likely pathogenic according to ACMG criteria, specifically applying the ClinGen ACADVL VCEP specifications. Conclusions: This study expands the *ACADVL* mutational spectrum and underscores the value of integrating sequencing, segregation, and structural bioinformatics in interpreting rare variants detected through NBS.

## 1. Introduction

Very long-chain acyl-CoA dehydrogenase deficiency (VLCADD; OMIM 201475) is a rare autosomal recessive disorder of mitochondrial fatty acid β-oxidation (FAO) [1]. It results from variants in the *ACADVL* gene, which encodes the enzyme very long-chain acyl-CoA dehydrogenase (VLCAD). VLCAD catalyzes the first and rate-limiting dehydrogenation step of long-chain acylcarnitine (LCA) (C14–C20) within the inner mitochondrial membrane. The resulting acyl-CoA derivatives enter subsequent β-oxidation cycles, producing acetyl-CoA, which feeds into the tricarboxylic acid (TCA) cycle and hepatic ketogenesis, thereby supporting cellular energy needs during fasting or metabolic stress [2].

The clinical consequences of impaired VLCAD function are severe and multifactorial, arising from the accumulation of toxic LCA and the deficiency of ketone bodies and ATP. These pathophysiologic mechanisms lead to mitochondrial dysfunction, including oxidative stress, swelling, and apoptosis in energy-demanding tissues such as the myocardium, liver, and skeletal muscle [3].

VLCADD presents with variable severity and age of onset, spanning from the neonatal period to adulthood. Based on predominant tissue involvement and age at onset, three major phenotypes are recognized: (a) a severe early-onset cardiac form, typically manifesting in infancy with hypertrophic or dilated cardiomyopathy, arrhythmias, respiratory distress, and high mortality associated with hypoketotic hypoglycemia and multiorgan failure; (b) an intermediate hepatic form, arising in late infancy or early childhood, characterized by recurrent episodes of fasting-induced hypoketotic hypoglycemia and liver dysfunction, often precipitated by infections, with less frequent cardiac involvement; (c) a late-onset myopathic form, presenting during adolescence or adulthood, typically with exertional rhabdomyolysis, myalgia, and myoglobinuria, without hypoglycemia or cardiac manifestations [4].

Despite the variability in presentation, all forms share a risk of life-threatening metabolic decompensation during catabolic stress, underscoring the importance of early diagnosis and metabolic management. Current therapeutic strategies focus on dietary modulation to reduce metabolic stress. Patients are typically managed with high-carbohydrate and low-fat regimens, restriction of long-chain fatty acids, supplementation with medium-chain triglycerides, strict avoidance of fasting, and careful monitoring to maintain stable blood glucose and adequate energy supply. Symptomatic management and treatment of complications remain essential adjuncts to this preventive approach [5]. The degree of residual VLCAD enzymatic activity often correlates with clinical severity: <10% activity is commonly associated with the severe neonatal form, while >30% may result in mild or asymptomatic presentations [6].

The global incidence of VLCADD is estimated to be between 1 in 30,000 and 1 in 100,000 live births [7]. However, prevalence varies significantly by population and diagnostic approach.

The hallmark biochemical indicator is elevated levels of LCA, particularly C14:1, in dried blood spots [4,8]. However, the biochemical signature alone has limited specificity, with high rates of false positives and variable overlap between heterozygous carriers and affected individuals. Confirmatory testing using plasma acylcarnitines, urine organic acids, enzyme activity assays, and *ACADVL* sequencing is essential to establish the diagnosis and guide management.

The *ACADVL* gene is located on chromosome 17p13.1 and spans approximately 5.4 kb. It consists of 20 exons and encodes a 655-amino acid protein with structural and functional domains essential for electron transport and catalysis. VLCAD is localized to the inner mitochondrial membrane, where it functions as a homodimer [9]. The N-terminal region of VLCAD contains the FAD-binding domain, while the C-terminal domain mediates membrane association and interaction with electron transfer flavoprotein (ETF). Electrons from VLCAD are shuttled through ETF to ETF dehydrogenase (ETFDH), eventually reaching coenzyme Q in the electron transport chain (ETC), linking FAO with oxidative phosphorylation (OXPHOS) [2].

Missense mutations that affect these domains may compromise proper folding, membrane integration, or electron transfer, thereby exacerbating mitochondrial dysfunction beyond energy deficit. Over 500 pathogenic and likely pathogenic variants have been described in the *ACADVL* gene, including missense, nonsense, small deletions/insertions, and splice-site alterations. Some variants are population-specific or founder mutations, while others appear sporadically. Null variants are typically associated with complete absence of VLCAD activity and severe neonatal phenotypes, whereas missense variants often allow residual activity and associate with intermediate or mild phenotypes.

One of the most frequently observed pathogenic variants is c.848T>C (p.Val283Ala), which accounts for approximately 29% of all cases. Although p.Val283Ala does not affect the catalytic or FAD-binding sites, it is located in a structurally important external loop near the periphery of the VLCAD protein, where it likely compromises protein stability rather than catalytic function. In vitro studies have shown that this variant is associated with residual enzymatic activity of 20–25%, and biochemical profiles consistently show elevated levels of the VLCADD marker C14:1. The allele is thought to be of European origin, supported by its high prevalence among Caucasian individuals in U.S. cohorts [10].

Phenotypic expression in individuals heterozygous for p.Val283Ala in trans with another pathogenic variant is highly variable, ranging from fatal neonatal hypoglycemia to asymptomatic adulthood. This variability has been attributed to the nature of the second allele: individuals with a null variant in trans often exhibit severe forms, whereas those with another missense variant may retain partial enzymatic function and present with milder phenotypes [11,12].

Here, we provide a comprehensive characterization of the *ACADVL* p.Ser72Phe (c.215C>T) variant in trans with p.Val283Ala, identified in a VLCADD patient through newborn screening (NBS).

## 2. Materials and Methods

Patients were recruited at the Medical Genetics Unit of the Renato Dulbecco University Hospital in Catanzaro, Italy. The study was conducted in accordance with the Declaration of Helsinki and institutional ethical standards and was approved by the “Comitato Etico Territoriale Regione Calabria” (Protocol n. 49/2025 of 27 February 2025). Written informed consent for genetic testing and data usage was obtained from all participants.

Genomic DNA was extracted from peripheral blood by using a commercial kit (Nuclear Laser Medicine, Settala, MI, Italy), according to the manufacturer’s instructions, and amplified by PCR using the forward and reverse primers binding exons 4 and 9 of the *ACADVL* gene reference sequence NM_000018.4. PCR products were bidirectionally sequenced using Big Dye Terminator 1.1 on a SeqStudio Genetic Analyzer (Thermo Fisher Scientific, Waltham, MA, USA).

The identified c.215C>T (p.Ser72Phe) variant was evaluated for pathogenicity using multiple bioinformatics tools: SIFT, PolyPhen-2, MutationTaster, CADD phred, REVEL, and AlphaMissense.

Pathogenicity was interpreted following the ACMG guidelines [13], applying the specifications of the ACMG/AMP guidelines for *ACADVL* variant interpretation [14].

To explore the structural impact of the p.Ser72Phe substitution, the crystal structure of human VLCAD (PDB ID: 2UXW; high-resolution 2.00 Å) was obtained from the Protein Data Bank, preserving its functional homodimeric assembly to capture the true biological context. Structural optimization and energy calculations were performed using the latest FoldX version 4.0 (build 20261231) under standard conditions (temperature 298 K, pH 7.0, implicit solvent model). The raw crystallographic coordinates were initially processed using the RepairPDB command to optimize torsion angles and resolve intrinsic structural strains. During this procedure, the essential FAD cofactor was explicitly retained within its binding pocket to maintain domain integrity, while crystallographic water molecules were removed to comply with the implicit solvation model parameters. The mutation was introduced using the FoldX BuildModel command across five independent runs (numberOfRuns = 5) to ensure statistical robustness. Total free energy (ΔG) was computed for both the wild-type and mutant structures, and the change in folding free energy (ΔΔG) was calculated as: ΔΔG = ΔG_mutant − ΔG_wild-type.

To further characterize conformational changes induced by the p.Ser72Phe variant, both the native and mutant VLCAD models were analyzed using UCSF ChimeraX (version 1.7). Hydrogen bonds and close contacts were assessed using the “findhbond” and “contacts” commands with default geometric criteria (donor–acceptor distance ≤ 3.5 Å; hydrogen–acceptor angle ≥ 120°). Newly formed interactions involving Phe72 were manually inspected and measured using the built-in distance tool in ChimeraX.

## 3. Results

In a full-term male neonate (III.3) born to unrelated Italian parents, liquid chromatography–tandem mass spectrometry (LC–MS/MS) analysis of acylcarnitines performed on dried blood spots at the end of the first postnatal week revealed a markedly abnormal biochemical profile. The diagnostic marker C14:1 reached 1.837 μmol/L, approximately five times above the upper reference limit, and other long-chain acylcarnitines were also elevated. According to external reports, the proband subsequently developed hypoketotic hypoglycemia, consistent with VLCADD. The proband is currently 5 years old; however, detailed longitudinal follow-up data were not available to our group.

The diagnosis of VLCAD deficiency was then confirmed by Sanger sequencing performed at CEINGE-Biotecnologie Avanzate Franco Salvatore S.C.A R.L of Naples. Genetic analysis revealed compound heterozygosity for two *ACADVL* variants: c.848T>C (p.Val283Ala) and c.215C>T (p.Ser72Phe) in trans position. The c.848T>C variant is a missense variant predicted to cause substitution of valine by alanine at position 283 and it is considered a common pathogenic variant associated with VLCAD deficiency [15]. The c.215C>T (p.Ser72Phe) variant is a missense variant not well characterized in the literature; however, SIFT, PolyPhen-2, and MutationTaster predicted a damaging role, while REVEL and CADD-Phred established the probable pathogenicity of the variant with scores of 0.941 and 30.00, respectively. AlphaMissense further classified the variant as likely pathogenic with a score of 0.973. This variant, cataloged as rs1161495077 and initially classified as a Variant of Uncertain Significance (VUS) (PM2_supporting, PP3 and PP4), has a very low minor allele frequency (MAF) of 0.000001239 in the gnomAD v4.1.1 database (also checked across European non-Finnish subsets) and alignment across multiple species demonstrated that the Ser72 residue is highly conserved, supporting its rarity and functional importance.

Genetic counseling and Sanger sequencing were also extended to the proband’s family members in our laboratory. The father (II.5), heterozygous for both p.Ser72Phe and p.Val283Ala, had a history of recurrent childhood hypoglycemia manifesting as syncope requiring glucose infusion, although he experienced normal psychomotor development. These episodes mirror the clinical history of his affected son, underscoring the pathogenic potential of the variant combination. The mother (II.6) carried p.Val283Ala in heterozygosity and reported bronchial asthma, acute bronchopneumonia at age two, and postpartum uterine cysts treated with dienogest. One paternal aunt (II.2), compound heterozygous for the same proband’s variants, had experienced a collapse at 10 months following febrile illness, persistent abdominal pain, and recurrent exertional intolerance leading to near-syncope episodes suggestive of mild rhabdomyolysis. Another paternal aunt (II.3) was heterozygous for p.Ser72Phe and has a history of hypothyroidism treated with levothyroxine, while a further aunt (II.4) was wild-type for both alleles and is currently managed for hyperthyroidism. The proband’s elder brother (III.2) was homozygous for p.Val283Ala and was also diagnosed through NBS, though no additional clinical data were available. The only information documented concerns his C14:1 level, measured at 0.409 μmol/L, which is above the reference threshold but not markedly elevated. The cousin (III.1), reported unexplained limb pain over the past year. The paternal grandfather (I.1), a heterozygous carrier of p.Val283Ala, has hyperthyroidism and diabetes mellitus, while the paternal grandmother (I.2), heterozygous for p.Ser72Phe, has Hashimoto’s thyroiditis, hypothyroidism, rheumatoid arthritis, and diabetes mellitus, conditions considered strictly incidental and entirely unrelated to the ACADVL genetic background.

Segregation analysis across three generations confirmed the inheritance of both alleles and demonstrated variable expressivity ranging from asymptomatic carriers to individuals with hypoglycemia, exertional intolerance, or metabolic crises. Moreover, the segregation data provided additional evidence fulfilling the PP1 and PM3 criteria, which, when applied according to the ACMG/AMP specifications refined for *ACADVL* variant interpretation [14], enabled the reclassification of c.215C>T (p.Ser72Phe) from a VUS to a likely pathogenic variant (PP3, PP1_moderate, PP4, PM2_supporting, PM3; see Supplementary Table S1 for the comprehensive walkthrough of each applied criterion and the relative evidence).

**Figure 1:**
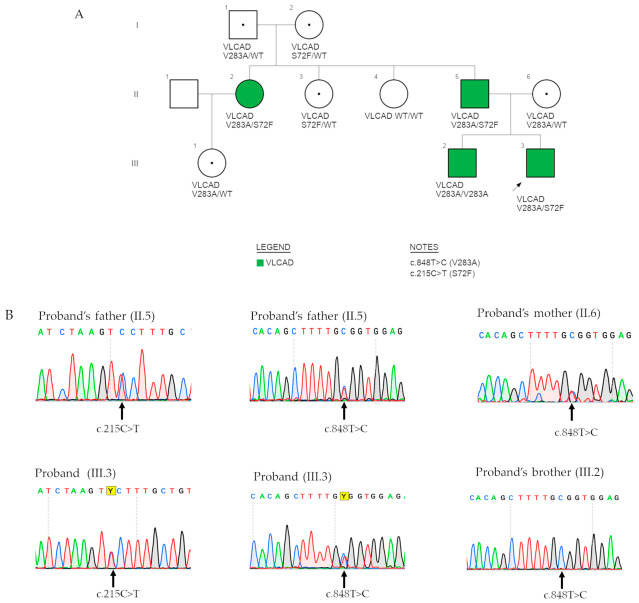
(**A**) Pedigree of the family with VLCAD deficiency. Squares indicate males and circles indicate females. Green-filled symbols represent affected individuals, open symbols represent unaffected individuals, and carriers are indicated by a black dot within the symbol. The proband (III.3) is indicated by an arrow. V283A corresponds to the *ACADVL* pathogenic variant c.848T>C (p.Val283Ala), S72F corresponds to the *ACADVL* likely pathogenic variant c.215C>T (p.Ser72Phe), and WT indicates wild-type alleles. (**B**) Representative Sanger sequencing chromatograms. The proband (III.3) and his father (II.5) are compound heterozygous for c.848T>C (p.Val283Ala) and c.215C>T (p.Ser72Phe). The proband’s mother (II.6) is heterozygous for c.848T>C (p.Val283Ala), while the proband’s brother (III.2) is homozygous for c.848T>C (p.Val283Ala).

The pedigree (Figure 1A) illustrates the inheritance pattern of the identified *ACADVL* variants, while Figure 1B shows the chromatograms of the proband’s immediate family (mother, father, and brother); chromatograms of the extended family are provided in Supplementary Figure S1.

The FoldX 4.0 analysis of the VLCAD structure (PDB ID: 2UXW) performed across five independent runs on the repaired homodimer produced a mean change in folding free energy (ΔΔG) of +22.63 ± 5.48 kcal/mol, indicating a significant localized thermodynamic burden. Energy decomposition revealed that van der Waals clashes contributed +20.85 ± 6.45 kcal/mol, sidechain hydrogen bonding +2.93 ± 0.45 kcal/mol, and backbone hydrogen bonding +1.48 ± 0.51 kcal/mol, whereas solvation hydrophobic terms provided a stabilizing contribution of −3.97 ± 0.17 kcal/mol.

**Figure 2:**
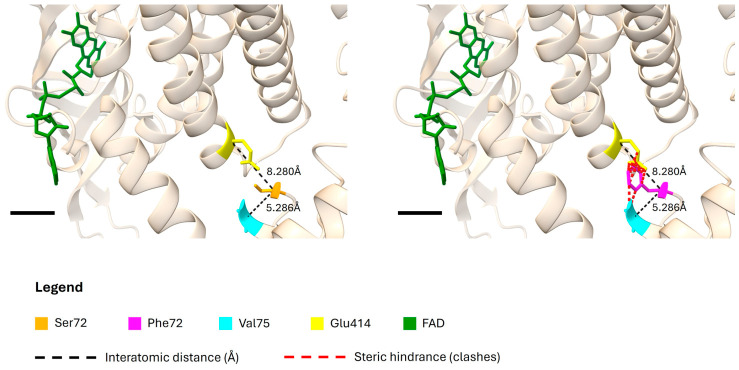
Structural modeling of the VLCAD wild-type (WT, **left**) and mutant p.Ser72Phe (**right**) proteins. The WT structure shows the native microenvironment surrounding residue Ser72, where interatomic distances from Glu414 (8.3 Å) and Val75 (5.3 Å) to the α-carbon are maintained without steric hindrance. The Ser72Phe substitution introduces a bulky aromatic side chain that alters local packing. While the backbone position remains unchanged, the mutant residue forms unfavorable short-range steric contacts (clashes, highlighted as red disks) with nearby residues, potentially reshaping the FAD-binding domain microenvironment by attenuating the native polar network.

Visual inspection using UCSF ChimeraX revealed that the Phe72 side chain introduces increased steric bulk and short-range van der Waals/CH-π-type contacts with nearby residues (including Val75 and the side-chain region of Glu414), coupled with partial attenuation of the hydrogen-bonding network normally contributed by Ser72. These contacts, clearly visible in Figure 2 and absent in the wild-type configuration, slightly distort the FAD-binding microenvironment and may hinder efficient cofactor positioning.

## 4. Discussion

This study presents a comprehensive characterization of the p.Ser72Phe variant in the *ACADVL* gene, identified in compound heterozygosity with the common p.Val283Ala allele in a patient diagnosed with VLCADD through newborn screening. By integrating clinical, molecular, bioinformatic, and structural modeling data, we provide convergent evidence supporting the pathogenic nature of this variant.

Bioinformatic predictions uniformly classified the p.Ser72Phe substitution as deleterious. Conservation analysis underscored the importance of residue Ser72, which lies within the FAD-binding domain, a region highly sensitive to amino acid perturbations. The multi-run FoldX calculation performed on the repaired homodimeric complex indicated a mean change in folding free energy (ΔΔG) of +22.63 ± 5.48 kcal/mol, corresponding to a significant localized thermodynamic burden. The energetic decomposition showed that van der Waals repulsion and torsional strain were the main contributors to destabilization, while hydrophobic solvation partially counterbalanced this effect. These data suggest that the mutation induces local structural strain and perturbs optimal sidechain packing rather than causing large-scale unfolding of the enzyme.

ChimeraX visualization further supported this interpretation by showing increased steric crowding and reduced hydrogen-bond stabilization near the FAD-binding pocket. The phenylalanine substitution replaces a polar residue with a bulky hydrophobic sidechain, disrupting the fine-tuned network that orients the cofactor. Such alterations are expected to compromise FAD binding affinity or electron transfer kinetics, consistent with previous observations that subtle structural distortions in VLCAD can affect mitochondrial function and OXPHOS coupling [2].

The p.Ser72Phe variant was previously reported in a Chinese patient with severe infantile VLCADD who died in early life, carrying this substitution in a compound heterozygous state with the p.Arg450His mutation [16]. In contrast, our patient carrying p.Ser72Phe in trans with p.Val283Ala currently appears to exhibit a less severe clinical presentation, although long-term follow-up data are not available. This phenotypic discrepancy is explained by the fact that the p.Val283Ala variant is traditionally known to retain residual VLCAD activity, thereby likely mitigating the impact of p.Ser72Phe. Such a divergence beautifully illustrates the profound impact of allelic context, residual enzyme activity, and possibly modifier genes on clinical outcome. This hypothesis of a milder clinical impact aligns precisely with the family’s clinical history, as the two compound heterozygous adults carrying the identical p.Ser72Phe variant remained entirely undiagnosed until adulthood, when targeted cascade testing was initiated only following the identification of the affected proband. Furthermore, the high ΔΔG value obtained in silico must be framed within this specific biological context: the severe energetic penalty is strictly localized near the cofactor-binding pocket. This localized thermodynamic burden allows for the hypothesis that the overall dimeric scaffold remains partially functional, leaving sufficient room for residual enzymatic efficiency that perfectly aligns with the non-severe clinical presentation observed in our proband.

Recent work has further emphasized that genotype–phenotype correlations in VLCADD are often blurred by metabolic flexibility, environmental influences, and genetic background, highlighting the value of integrative approaches that combine genomics, metabolomics, and computational modeling to improve variant interpretation [17]. Our study echoes this paradigm, as we combine sequencing data with structural bioinformatics to refine the classification of a rare missense allele.

Another relevant consideration is the concept of a “spectrum of functional impairment” in VLCAD deficiency, where mutations are not strictly pathogenic or benign, but instead produce gradual shifts in enzymatic efficiency that modulate clinical expression [17]. The compound heterozygous state observed here—p.Ser72Phe and p.Val283Ala—likely reduce VLCAD function below a clinical threshold, while residual activity and early nutritional intervention preserve metabolic stability.

With the currently available data, some hypothetical considerations can be made: referring back to the study conducted in the USA [10], patients harboring two pathogenic variants generally displayed C14:1 levels >1 μmol/L; conversely, almost all patients with zero or one variant had levels below this threshold, with approximately 62% showing values below the upper limit of normal (C14:1 < 0.196 μmol/L). Considering that the proband’s NBS analysis revealed a C14:1 concentration of 1.837 μmol/L, it is plausible that the p.Ser72Phe variant significantly contributes, in conjunction with the p.Val283Ala, to the elevation of acylcarnitine levels. This hypothesis is further supported by the observation that the proband’s genotype is associated with higher metabolite levels compared with his brother, who does not carry p.Ser72Phe.

In this context, it can be hypothesized that individuals carrying the p.Ser72Phe variant in trans with a missense mutation (or at most small in-frame deletions or insertions) belong to the category of VLCAD patients with a mild clinical phenotype, given the missense nature of p.Ser72Phe. Supporting this notion, outcomes of individuals harboring a pathogenic allele in trans with p.Val283Ala have shown considerable phenotypic heterogeneity: while severe neonatal hypoglycemia has been reported in association with a null allele in trans, a much milder course was observed in an individual who remained asymptomatic until the age of 30, when the second allele was missense, retaining modest residual enzymatic activity [10]. This assumption is consistent with the clinical course of the family described here, who, despite certain complications and necessary precautions, enjoy relatively good health. Although the p.Ser72Phe variant does not reside in the catalytic site, it appears that likely alterations in subunit interactions could impair VLCAD functionality, potentially exacerbating protein dysfunction when present in trans with other pathogenic alleles.

Taken together, our findings support the pathogenicity of p.Ser72Phe, though definitive biochemical confirmation remains necessary. We acknowledge as a primary limitation of this study the absence of direct functional validation, such as enzyme activity assays or expression studies in patient-derived fibroblasts or lymphoblastoid cell lines. Consequently, our findings regarding the disruption of subunit interactions, FAD binding affinity, or electron transfer kinetics must be interpreted as computational hypotheses rather than established experimental facts. Furthermore, our reliance on static structural models cannot fully capture the complex conformational dynamics of the protein, and the study is inherently constrained by the lack of a long-term clinical follow-up for the newer generation. Future studies employing molecular dynamics, cryo-EM structural refinement, and targeted in vitro functional assays will be crucial to validate these predictions.

In conclusion, this work expands the mutational spectrum of *ACADVL* and provides novel mechanistic insight into the underexplored p.Ser72Phe allele. By integrating classical genetics, structural bioinformatics, and clinical observation, we move toward a more nuanced interpretation of VLCADD variants, in line with current precision medicine approaches. Such integrative strategies may ultimately enable individualized risk stratification and targeted therapeutic interventions for patients with FAO disorders.

## Data Availability

The data that support the findings of this study are available on request from the corresponding author. The data are not publicly available due to privacy or ethical restrictions.

## Supplementary Materials

The following supporting information can be downloaded at: https://www.mdpi.com/article/10.3390/genes17060649/s1, Supplementary Table S1. ACMG Classification of c.215C>T (p.Ser72Phe) variant following ClinGen ACADVL Expert Panel Specifications to the ACMG/AMP Variant Interpretation Guidelines for ACADVL Version 2.1.0. Supplementary Figure S1. Representative Sanger sequencing chromatograms of extended family members. The proband’s grandfather (I.1) and cousin (III.1) are heterozygous for the ACADVL pathogenic variant c.848T>C (p.Val283Ala). The proband’s grandmother (I.2) is heterozygous for the ACADVL likely pathogenic variant c.215C>T (p.Ser72Phe). The proband’s paternal aunts show heterozygosity: individual II.2 for c.848T>C (p.Val283Ala), and individual II.3 for c.215C>T (p.Ser72Phe).

## Abbreviation

VLCADD	very long-chain acyl-CoA dehydrogenase deficiency
FAO	fatty acid β-oxidation
VLCAD	very long-chain acyl-CoA dehydrogenase
LCA	long-chain acylcarnitine
TCA	tricarboxylic acid
ETF	electron transfer flavoprotein
ETFDH	ETF dehydrogenase
ETC	electron transport chain
OXPHOS	oxidative phosphorylation
NBS	newborn screening
LC–MS/MS	liquid chromatography–tandem mass spectrometry
VUS	variant of uncertain significance
MAF	minor allele frequency
