# Supplementary material for: Expanding the Mutational Spectrum of ACADVL: Integrative Characterization of the p.Ser72Phe Variant in Very Long-Chain Acyl-CoA Dehydrogenase Deficiency
Source: Genes (Basel). 2026 May 31;17(6):649. doi: 10.3390/genes17060649 (PMC13299349; doi:10.3390/genes17060649)
Supplement: Supplementary file 1 [file genes-17-00649-s001.zip › Supplementary Figure S1.pdf]

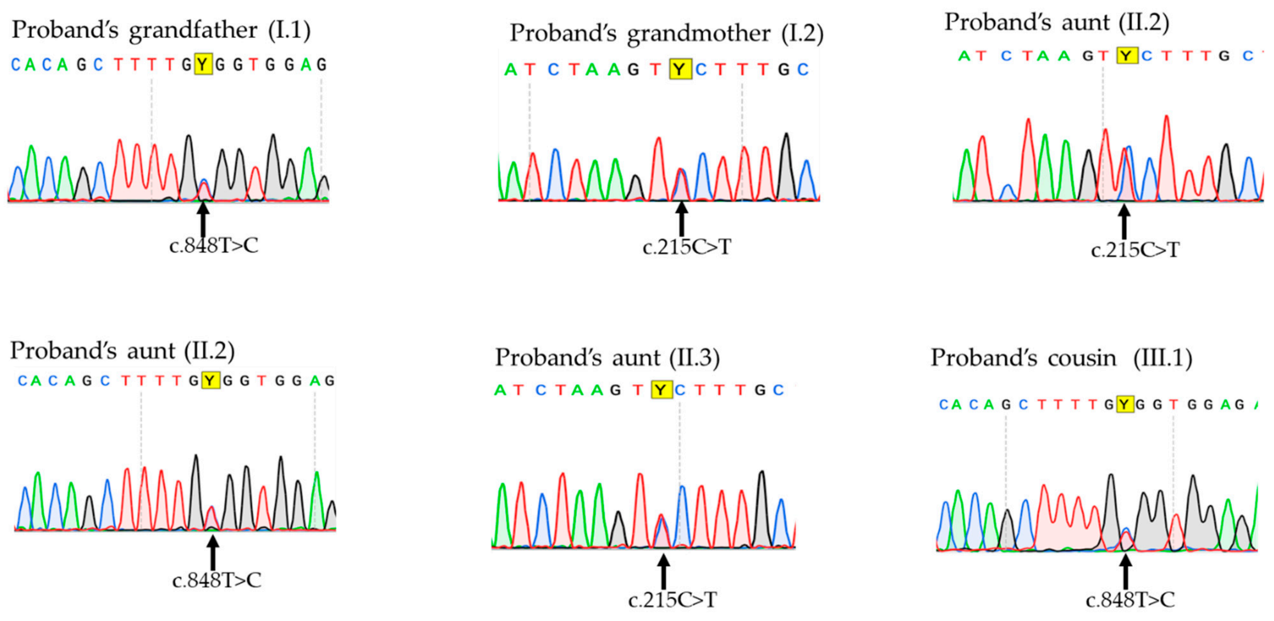

Supplementary Figure S1. Representative Sanger sequencing chromatograms of extended family members. The proband's grandfather (I.1) and cousin (III.1) are heterozygous for the ACADVL pathogenic variant c.848T>C (p.Val283Ala). The proband's grandmother (I.2) is heterozygous for the ACADVL likely pathogenic variant c.215C>T (p.Ser72Phe). The proband's paternal aunts show heterozygosity: individual II.2 for c.848T>C (p.Val283Ala), and individual II.3 for c.215C>T (p.Ser72Phe).
