# Supplementary material for: Expanding the Mutational Spectrum of ACADVL: Integrative Characterization of the p.Ser72Phe Variant in Very Long-Chain Acyl-CoA Dehydrogenase Deficiency
Source: Genes (Basel). 2026 May 31;17(6):649. doi: 10.3390/genes17060649 (PMC13299349; doi:10.3390/genes17060649)
Supplement: Supplementary file 1 [file genes-17-00649-s001.zip › Supplementary Table S1.pdf]

Supplementary Table S1. ACMG Classification of c.215C>T (p.Ser72Phe) variant following ClinGen ACADVL Expert Panel Specifications to the ACMG/AMP Variant Interpretation Guidelines for ACADVL Version 2.1.0.

**Criterion**	**Strength**	**Evidence**
PM3	Moderate	Variant confirmed *in trans* with p.Val283Ala in the proband.
PP1	Moderate	Two affected segregations (II.2 and II.5) and two unaffected segregations (II.3 and II.4). Proband and grandparents were excluded. LOD score < 1.50 ≥ 1.20 (1.45)
PM2	Supporting	Allele frequency in gnomAD v4.1.1 <0.001 (0.000001239)
PP4	Supporting	C14:1 Acylcarinitine value > 0.8 µM/L (1.837)
PP3	Supporting	REVEL score >0.75 (0.941)
